# Linking the bacterial microbiome between gut and habitat soil of Tibetan macaque (*Macaca thibetana*)

**DOI:** 10.1002/ece3.9227

**Published:** 2022-09-13

**Authors:** Xiaojuan Xu, Yingna Xia, Binghua Sun

**Affiliations:** ^1^ School of Life Science Hefei Normal University Hefei China; ^2^ School of Resource and Environmental Engineering Anhui University Hefei China; ^3^ International Collaborative Research Center for Huangshan Biodiversity and Tibetan Macaque Behavioral Ecology Anhui University Hefei China

**Keywords:** gut bacterial microbiome, mammals, soil bacterial microbiome, Tibetan macaque

## Abstract

Soil is a part of the habitat environment of terrestrial or semi‐terrestrial mammals, which contains a wide variety of microbes. Although the soil microbiome of the host habitat is considered to be a potentially important influence factor on the mammalian gut microbiome and health, few data are currently available to explore the relationship between gut and host habitat soil microbiome in wild primates. Here, marked divergence of the bacterial microbiome in composition and structure between Tibetan macaques (*Macaca thibetana*) guts and its habitat soil were detected. In addition, we found that most of the core genera abundance and ASVs in the Tibetan macaques' gut bacterial microbiome could be detected in the corresponding soil samples, but with low abundance. However, the core abundant genera abundant in soil are almost undetectable in the gut of Tibetan macaques. Although there are some ASVs shared by gut and soil bacterial microbiome, the abundant shared ASVs in the guts of Tibetan macaques were rare bacterial taxa in the corresponding soil samples. Notably, all the ASVs shared by guts and soil were present in the soil at relatively low abundance, whereas they were affiliated with diverse bacterial taxa. By linking the bacterial microbiome between Tibetan macaques’ gut and its habitat soil, our findings suggest that the predominant bacterial groups from the soil were not likely to colonize the Tibetan macaques' gut, whereas the low‐abundance but diverse soil bacteria could be selected by the gut. Whether these rare and low‐abundant bacteria are permanent residents of the soil or a source of fecal contamination remains to be determined in future study.

## Graphical abstract

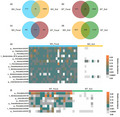

## 1 INTRODUCTION

The gut microbiome is considered to be a functional “organ” of mammals (O'Hara & Shanahan, 2006), which plays a crucial role in host nutrition, immune systems, development, and health (Flint et al., 2012; Fung et al., 2017; Nicholson et al., 2012; Sommer & Backhed, 2013). The colonization of the mammalian gut microbiome starts at birth, with microbial diversity continuing to increase, influenced by endogenous and exogenous factors (Dreyer & Liebl, 2018). Mounting studies had revealed many factors, including host genetics, physiology, behavior, diet, and group size and composition affect the composition, structure, and stability of the host gut microbiome (Linnenbrink et al., 2013; Suzuki et al., 2019; Tung et al., 2015; Zmora et al., 2019), while the microbes of host's habitat environment also act to affect the community composition of the mammalian gut microbiome (Seedorf et al., 2014; Smith et al., 2015). Explore the potential relationship between the mammalian gut microbiome and its habitat environmental microbiome is critical to understanding the processes of gut microbiome assembly and differences in the gut ecosystem among hosts living in different environments (Tasnim et al., 2017).

Soil is a part of the habitat environment of humans and other terrestrial mammals, which provides mammals with space for living, social activities, and food production. One of the important characteristics of soil is that it contains the most diverse and abundant group of microbes on Earth, and plays an important role in primary productivity and nutrient cycling (Fierer, 2017; Torsvik & Øvreås, 2002). Terrestrial and semi‐terrestrial animals' hands, feet, and fur are often in contact with soil, as well as the plant diets are close to the ground. This provides an opportunity for soil microbes to enter the gut of animals through feeding behavior (Grieneisen et al., 2019). Some evidence suggested that mammalian gut microbiome and health are potentially affected by the soil microbes of their habitats (Blum et al., 2019; Wall et al., 2015). For example, soil biodiversity provides benefits to the human microbiome (Hanski et al., 2012), as well as provides “natural immunity” (von Hertzen et al., 2011). Exposure to soil microbes has been reported to increase gut microbiota diversity of lab mice and piglets (Vo et al., 2017; Zhou et al., 2016, 2018). Unexpectedly, in humans, Tasnim et al. (2017) reported that soil and gut bacterial communities have few overlapping bacterial taxa (Tasnim et al., 2017). Recently, a study on Plateau pikas (*Ochotona curzoniae*) and Daurian pikas (*Ochotona. daurica*) suggested that the gut may mainly select for low‐abundance but diverse soil bacteria (Li et al., 2016). Further studies in a wider range of species are needed to clarify the relationship between the mammalian gut microbiome and their habitat soil microbiome.

Nonhuman primates (NHPs) share broadly similar morphological, physiological, and genetic characteristics with humans. NHPs are important animal model systems for understanding many aspects of human behavior, cognition, physiology, and health, as well as the gut microbiome (Clayton et al., 2018; Phillips et al., 2015). Previous studies on the gut microbiome of terrestrially living baboons (*Papio cynocephalus* and *Papio anubis*) showed that soil is the most dominant predictor for shaping the gut microbiota with a 15 times stronger effect than host genetics (Grieneisen et al., 2019). Soil microbes are also considered to be a potentially important factor to explain that sympatric terrestrial *Pan* and *Gorilla* share more bacterial taxa than those from disparate regions (Moeller et al., 2016). In addition, the gut microbiomes of arboreal species Verreaux's sifaka (*Propithecus verreauxi*) and red‐tailed sportive lemur (*Lepilemur ruficaudatus*) are far more differences than those of their terrestrial and semi‐terrestrial counterparts, such as semi‐terrestrial red‐fronted brown lemur (*Eulemur rufifrons*) and terrestrial cattle, bush pigs, and fossa (Perofsky et al., 2019). However, the current findings are mainly based on indirect evidence, the relationship between the wild NHPs' gut bacterial communities, and the habitat soil bacterial communities remain to be clarified.

Tibetan macaques (*Macaca thibetana*), a near‐threatened primate species endemic to China, is a semi‐terrestrial species of the genus *Macaca* (Li & Kappeler, 2020). To understand the associations of bacterial microbiome between the Tibetan macaques' gut and its habitat soil, we sequenced the macaques' gut bacterial communities at two different sites (Mt. Huangshan and Mt. Tianhu), as well as those from their surrounding environmental soil at each site. In the present study, we addressed the following three main objectives. First, we examined the differences of the bacterial microbiome in composition and structure between Tibetan macaques' gut and habitat soil. Second, to clarify the distribution pattern of the core abundant taxon of the gut bacterial microbiome in the soil samples. Lastly, to test whether there are shared microbes at amplicon sequence variants (ASVs) leveled between gut and soil bacterial microbiome, as well as the distribution pattern of the shared ASVs.

## 2 MATERIALS AND METHODS

### 2.1 Study subject and sample collection

This study was carried out at two sites in Anhui Province, China, including Mt. Huangshan (MH) and Mt. Tianhu (MT). The group living in Mt. Huangshan has been a behavioral research and ecotourism center since 1986. MH group is composed of 60 individuals and represents a free‐ranging group that is provisioned three times per day with a total of 5–6 kg of corn. The amount provisioned is approximately one‐third of the daily food intake of the group. Mt. Tianhu is located some 10 km from MH. MT group was discovered in 2018 and soon thereafter we began following and collecting data on this wild group. This group is composed of 91 macaques without food provision. Both sites share similar flora and fauna. The main diet of the MH and MT groups includes leaves and grass, and to a lesser extent, fruits, flowers, roots, and insects. All samples were collected during a 2‐week period in summer, from August 1 to 14, 2019. We obtained 46 fresh fecal samples of macaques in MH and MT (MH_Fecal: 27, MT_Fecal: 19) and 27 topsoil samples from the two field sites (MH_Soil: 14, MT_Soil: 13), each soil sample was a mixture of five individual soil cores at the depth of 0–10 cm, which randomly sample within 1 square meter, each soil sample was taken 10 meters apart. In total, we had 73 fecal and soil samples. All fecal samples were collected and placed in a sterilized sampling tube with RNAlater (QIA‐GEN, Valencia CA). Topsoil samples were placed into a sterilized polyethylene bag as a single composite sample. All the samples were placed in ice bags and transported to the laboratory at Anhui University within 12 h of collection, and stored at −80°C. This research was approved by the Institutional Animal Care and Use Committee of the Anhui Zoological Society (permit number AHZS201711008). All experiments were in accordance with their approved guidelines and regulations and complied with all principles of the China Animal Ethics Committee.

### 2.2 DNA extraction and sequencing

Total DNA from each soil sample was extracted using the FastDNA® Spin kit (Bio 101). To avoid soil contamination, the total DNA from feces was collected from the inner part of each fecal sample using a QIAamp® Fast DNA Stool Mini kit (Qiagen). The total DNA extracted from the 73 samples was sent to the Shanghai Majorbio Bio‐pharm Technology Co., Ltd. for sequencing. For each sample, the V3–V4 region of the 16S rRNA gene was amplified using primers 338F (5’‐ACTCCTACGGGAGGCAGCAG‐3′) and 806R (5’‐GGACTACHV GGGTWTCTAAT‐3′) (Mori et al., 2013). Reaction conditions were as follows: 94°C for 5 min, 94°C for 45 s × 30 cycles, 53°C for 45 s, 45 s at 72°C, and 10 min final extension at 72°C. PCR products were purified with a Min Elute PCR Purification kit (QIAGEN) and then quantified using the QuantiFluor‐ST and the dsDNA System (Promega). Purified amplicons were pooled in equal amounts, and pair‐end 2x300bp sequencing was performed on Illlumina Miseq platform at Shanghai Majorbio Bio‐pharm Technology Co., Ltd.

### 2.3 Bioinformatics and statistical analysis

We trimmed raw FASTQ sequencing data for the adaptor sequence and quality control using the sliding window approach implemented in fastp v0.19.6 (Chen et al., 2018). A window of 50 bp was set to filter the reads with a tail mass value of 20 or less. If the average mass value in the window was lower than 20, the rear bases were removed from the window, and the reads with a tail mass value of 50 bp after quality control were filtered. Those containing N bases were removed. We merged overlapping paired‐end reads using FLASH v1.2.7 (Magoč & Salzberg, 2011), with the minimum overlap set to 10 bp, the maximum error ratio of overlap area was 0.2, and the number of mismatches barcode allowed was 0. The maximum primer mismatch number was 2. Lastly, we clustered the quality check of sequences into amplicon sequence variants (ASVs) using DADA2 within Qiime 2 to truncate forward and reverse reads, denoise the data, and detect and remove chimeras (Bolyen et al., 2019; Callahan et al., 2016). Taxonomy was assigned to ASV using classify‐sklearn (Naive Bayes) with the database (v.132; https://www.arb‐silva.de/).

The Shannon diversity index (Shannon), ASV richness, and unweighted and weighted UniFrac distance matrices were calculated using Qiime 2 (Bolyen et al., 2019). We tested for normal distributions in alpha diversity indices, relative abundances of dominant phyla, and functional guilds using the Kolmogorov–Smirnov normality test. We used a one‐way ANOVA and Tukey's post hoc tests to test for differences across sample groups in case of a normal distribution, or a Kruskal–Wallis ANOVA with Dunn's multiple‐comparison test in cases of an abnormal distribution. *p*‐values were adjusted using a Bonferroni correction. Principal coordinates analysis (PCoA) was performed with the R packages Made4 and Vegan.3. Permutational multivariate ANOVA (PERMANOVA) was used to test for differences in beta diversity (unweighted and weighted UniFrac distance) using the Adonis function in the vegan R package (Chen et al., 2012). LEfSe (linear discriminant analysis effect size) was used with default options to determine genera enriched in each study group (Segata et al., 2012). In all analyses, the value of p was set at 0.05. The raw data were submitted to the Sequence Read Archive of NCBI under the accession number PRJNA739400.

## 3 RESULTS

### 3.1 General patterns of the bacterial profile

After bioinformatic processing, we obtained 2,233,921 high‐quality filtered reads. To eliminate the effects of different sequencing depths on the analyses, the data set was rarefied to 17,755 sequences per sample (the minimum sequence number among 73 samples). Taxonomic assignment revealed 43 phyla, 141 classes, 333 orders, 543 families, 1161 genera, and 26,278 ASVs. At the phylum level, the relative abundance of the unclassified bacteria was less than 1% in both fecal and soil samples. Only 10 phyla have an average relative abundance greater than 1% across all samples.

### 3.2 Composition of the gut and soil bacterial microbiome

**FIGURE 1:**
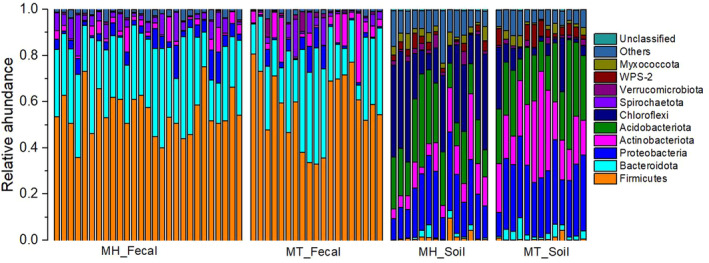
The distributions of phyla in different sample groups. Stacked bar graphs illustrate the abundances of phyla; the X‐axis represents the samples.

The dominant phyla across fecal samples were Firmicutes (x = mean ± Std. Deviation, x = 56.02 ± 12.05%), Bacteroidota (x = 28.02 ± 10.32%), Proteobacteria (x = 4.14 ± 3.72%), and Actinobacteriota (x = 4.59 ± 5.69%), whereas the soil samples were dominated by Proteobacteria (x = 23.82 ± 8.37%), Acidobacteriota (x = 22.40 ± 9.10%), Chloroflexi (x = 17.14 ± 15.13%), and Actinobacteriota (x = 16.57 ± 10.64%) (Figure 1a). At the family level, the fecal samples were dominated by Prevotellaceae (x = 19.42 ± 9.11%), Lachnospiraceae (x = 16.27 ± 9.71%), and Oscillospiraceae (x = 11.19 ± 4.66%), the soil samples were dominated by Xanthobacteraceae (x = 5.83 ± 2.33%), Ktedonobacteraceae (x = 3.44 ± 5.20%), and Solirubrobacteraceae (x = 3.27 ± 3.74%). In addition, the predominant known genera of fecal samples were *Prevotella*, *UCG‐005*, *Faecalibacterium*, *Treponema*, and *Succinivibrio*, whereas the soil samples were predominated by *Bryobacter*, *Candidatus_Solibacter*, *Acidothermus*, and *Conexibacter*.

**FIGURE 2:**
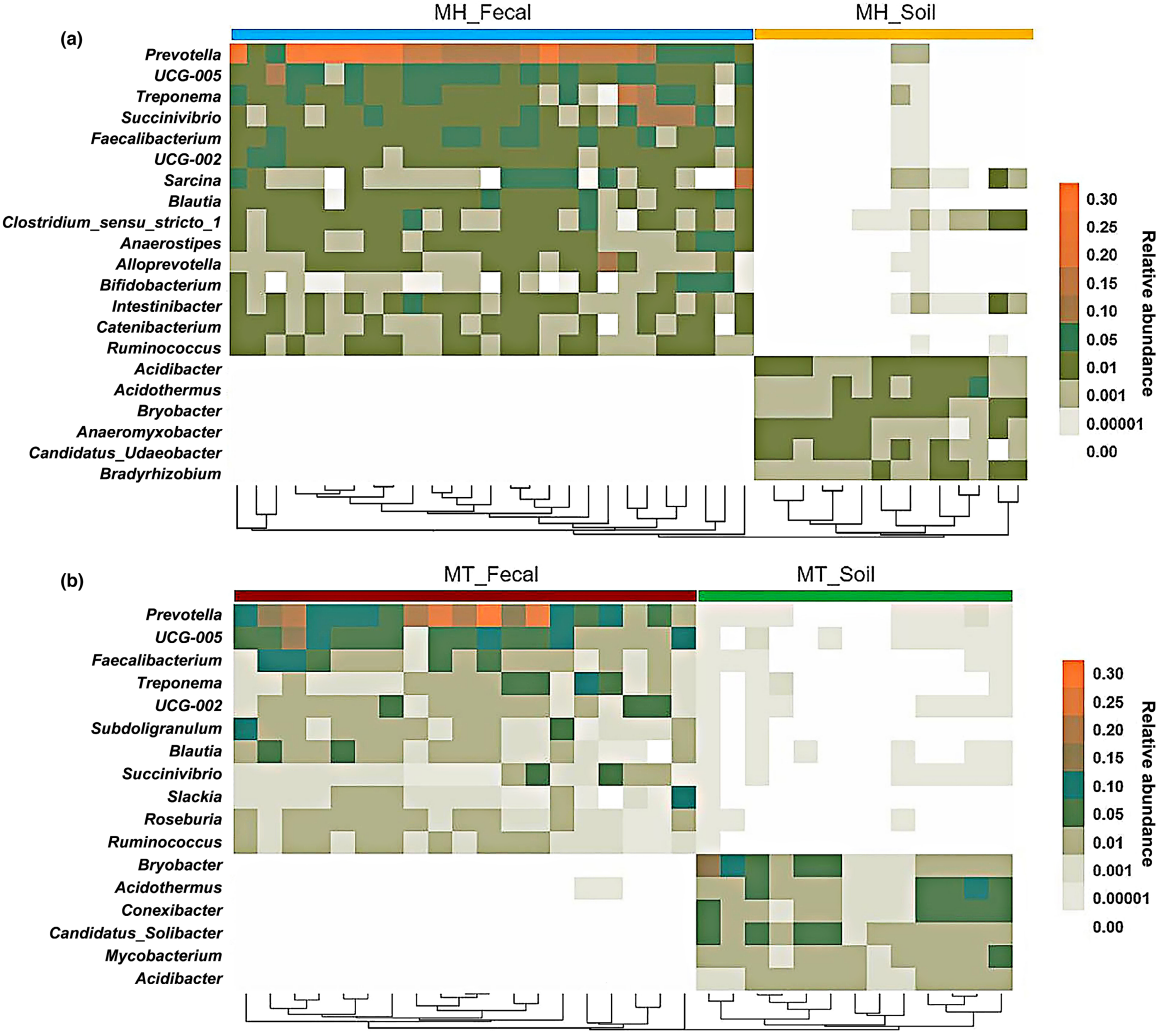
The distributions of core abundant known genera in fecal or soil samples. (a) The distributions of core abundant genera in fecal and soil samples of MH. (b) The distributions of core abundant genera in fecal and soil samples of MT. Core abundant known genera were defined as present on at least 80% of each sample type (fecal and soil) and at an average relative abundance of >1%. Heat maps were used to show the distribution patterns.

We defined core abundant known genera as present on at least 80% of each sample type (fecal and soil) and at an average relative abundance of >1%. Our results indicated the existence of 15 and 6 core abundant genera in fecal and soil samples of MH, respectively (Figure 2a). Similarly, we detected 11 and 6 core abundant genera in fecal and soil samples of MT, respectively (Figures 1 and 2). The taxonomic profiles, mean relative abundances, and occurrence rate of these genera were listed in Supplementary Table S1 and Table S2. The core genera abundance in the Tibetan macaques' gut bacterial microbiome was rarely present (low abundances: <1%, low occurrence rate: <80%) in the corresponding soil samples of MH and MT. Notably, the core abundant genera in soil samples were not detected in the fecal samples of MH at all. We also found that most of the core abundant genera in MT soil samples were also missing in the fecal samples, except for one core abundant genus (*Acidothermus*) that was rarely and only present in the corresponding two fecal samples (the relative abundance was <0.0001).

**FIGURE 3:**
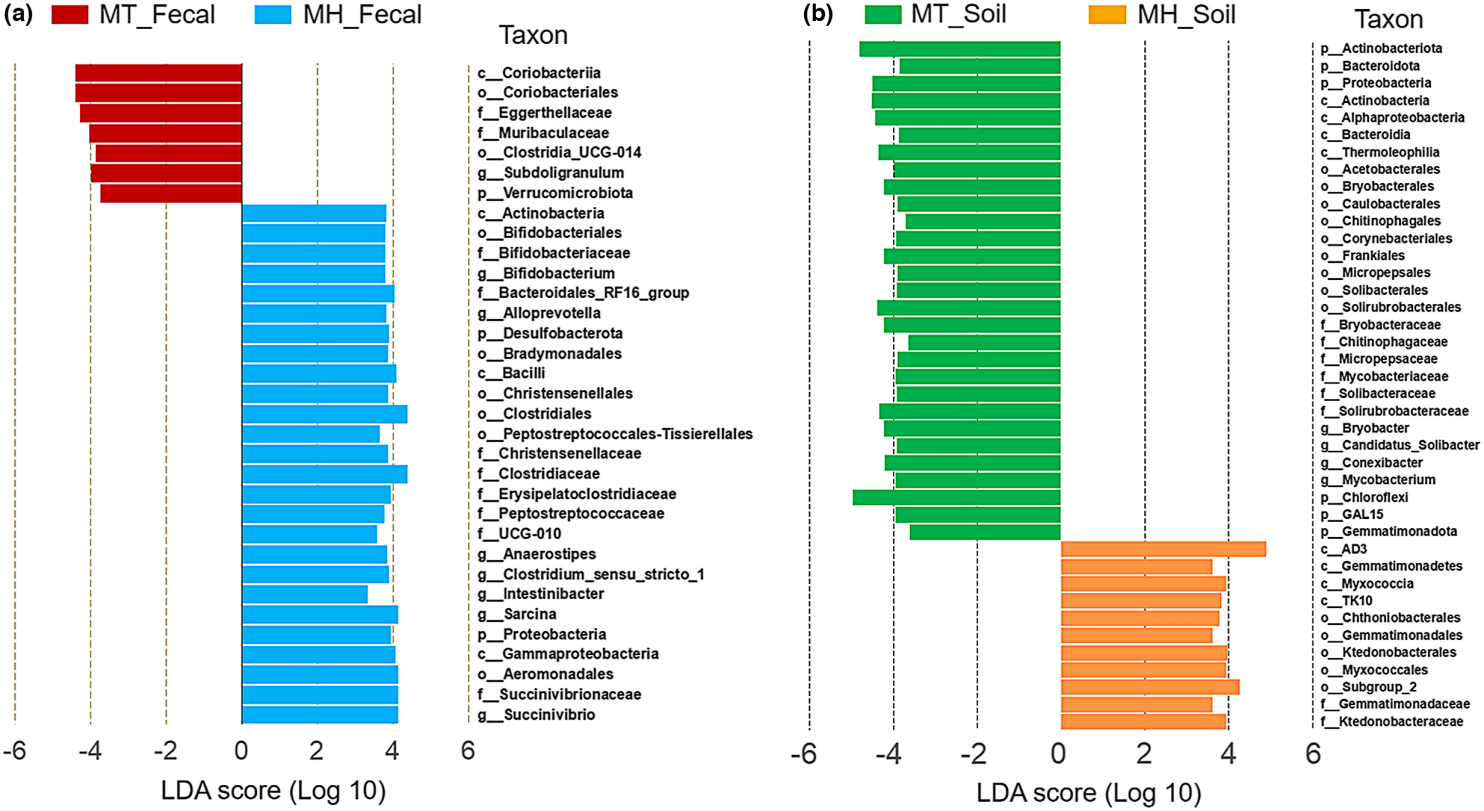
LEfSe analysis on the gut bacterial taxonomy. (a) Fecal samples between MH and MT groups. (b) Soil samples between MH and MT groups. Gut microbial taxonomy enriched in different reproductive states identified by LEfSe (LDA >3, *p* < .05).

LEfSe analyses revealed that each study population was characterized by different known bacterial taxa (at the genus, family, order, class, and phylum levels; the mean relative abundance of known taxa accounting for ≥1% of all the fecal samples). In total, 26 and 7 indicators were identified in MH and MT groups, respectively (LDA >3, *p* < .05; Figure 3a). At the genus level, *Subdoligranulum* was overrepresented in MT fecal samples, and seven other known genera, *Succinivibrio*, *Sarcina*, *Intestinibacter*, *Bifidobacterium*, *Anaerostipes*, *Clostridium_sensu_stricto_1*, and *Alloprevotella*, were overrepresented in the MH fecal samples. For the soil samples of both sites, 40 known taxa (at the genus, family, order, class, and phylum levels; the mean relative abundance of known taxa accounting for ≥1% of all the soil samples) were significantly enriched in one of the two soil sample groups (LDA >3, *p* < .05; Figure 3b). Among these taxa, 14 and 26 indicators were identified in MH and MT soil samples, respectively. At the genus level, four known genera *Mycobacterium*, *Conexibacter*, *Candidatus_Solibacter*, and *Bryobacter* were overrepresented in MT soil samples. However, neither genus was overrepresented in MH soil samples.

### 3.3 Diversity of the gut and soil bacterial microbiome

**FIGURE 4:**
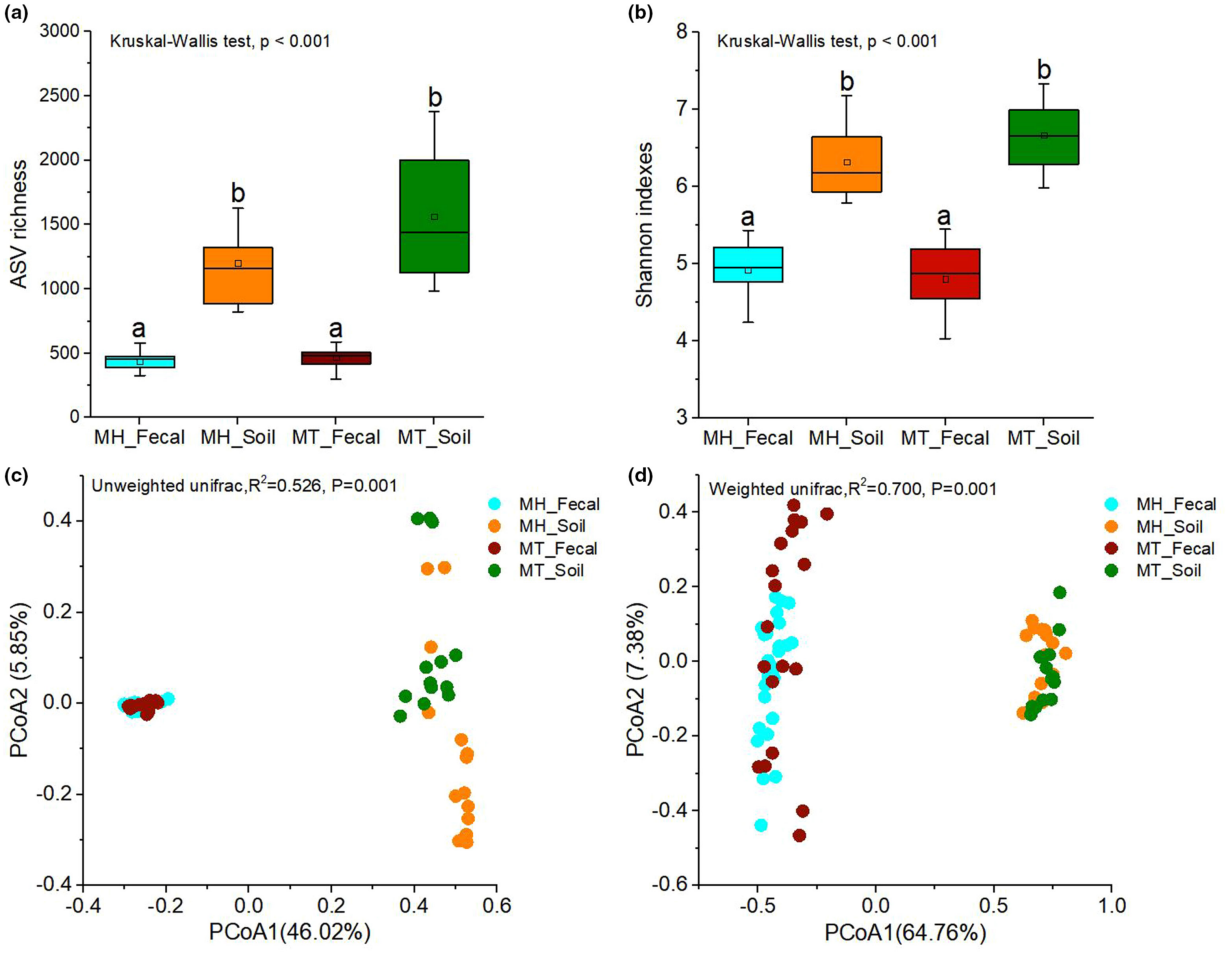
Differences in bacterial diversity across the sample groups. (a) Comparison of amplicon sequence variant (ASV) richness. (b) Comparison of Shannon diversity index. A Kruskal–Wallis ANOVA test was used to evaluate the variation across treatment groups. Post hoc tests (Dunn's test) for pairwise comparison tests (values of p were adjusted by Bonferroni). (c, d) Differentiation of bacterial microbiome structure (c) based on unweighted UniFrac distance and (d) based on weighted UniFrac distance. Principal coordinates analysis (PCoA) was used to show patterns across sample groups. Adonis tests were performed on unweighted and weighted UniFrac distances, respectively. Significance was set at the 0.05 level.

We calculated the Shannon diversity index (Shannon) and number of ASVs observed (ASV richness) of bacterial communities in Tibetan macaques and their habitat soil. We found that alpha diversity of bacterial microbiome was significant variation across sample groups, regardless of ASV richness or Shannon index (Kruskal–Wallis, ASV richness: df = 3, *F* = 51.973, *p* < .0001; Shannon: df = 3, *F* = 51.091, *p* < .001). Additional pairwise comparison analysis showed that the ASV richness of fecal samples was significantly lower than that of soil samples (adjusted *p* < .0001). No significant differences in ASV richness between the two sites of the same sample types (MH_Fecal vs. MT_Fecal, adjusted *p* = 1; MH_Soil vs. MT_Soil, adjusted *p* = 1) (Figure 4a,b). Next, we compared the Shannon index between any two groups. The result indicated that the Shannon index of fecal samples was significantly lower than the indices found for soil samples (adjusted *p* = .001). However, no significant difference was found between the two sites of the same sample types (MH_Fecal vs. MT_Fecal, adjusted *p* = 1; MH_Soil vs. MT_Soil, adjusted *p* = 1) (Figure 4b).

We performed PCoA and a PERMANOVA tests based on unweighted and weighted unifrac dissimilarities to investigate the variation in beta diversity in the bacterial microbiome across all samples from the two study sites. Our result revealed significant distinctions in bacterial microbiome profiles among all four sample groups (PERMANOVA, unweighted unifrac, *R* ^2^ = 0.526, *p* = 0.001; weighted unifrac, *R* ^2^ = 0.700, *p* = .001) (Figure 4c,d). Moreover, significant difference in beta diversity between same sample types also was detected based on unweighted unifrac dissimilarities (Adonis, MH_Fecal vs MT_Fecal: *R* ^2^ = 0.123, *p* = .001; MH_Soil vs MT_Soil: *R* ^2^ = 0.132, *p* = .001), and the same result also were detected based on weighted unifrac dissimilarities (Adonis, MH_Fecal vs. MT_Fecal: *R* ^2^ = 0.095, *p* = .002; MH_Soil vs. MT_Soil: *R* ^2^ = 0.194, *p* = .001).

### 3.4 The shared ASVs between gut and soil bacterial microbiome

We identified a total of 26,278 unique amplicon sequence variants (ASVs). In detail, 2443 and 24,069 ASVs were identified in fecal and soil samples, respectively. At Mt. Huangshan, there were 63 ASVs shared between fecal and soil samples, accounting for 3.52% (63 of 1780 total ASVs of MH_Fecal) and 0.58% (63 of 10,912 total ASVs of MH_Soil) of the total amount of ASVs in the corresponding feces and soil (Figure A). At Mt. Tianhu, there were 166 ASVs shared between fecal and soil samples, accounting for 9.32% (166 of 1781 total ASVs of MT_Fecal) and 1.12% (166 of 14,856 total ASVs of MT_Soil) of the total amount of ASVs in the corresponding feces and soil (Figure B). In addition, there were 1118 ASVs shared by MH and MT fecal samples, accounting for 62.81% and 62.77% of the total amount of ASVs in the corresponding samples. The shared ASVs of soil samples accounted for 15.57% and 11.44% of the total ASVs of MH and MT soil samples, respectively (Figure 3c,d).

**FIGURE 5:**
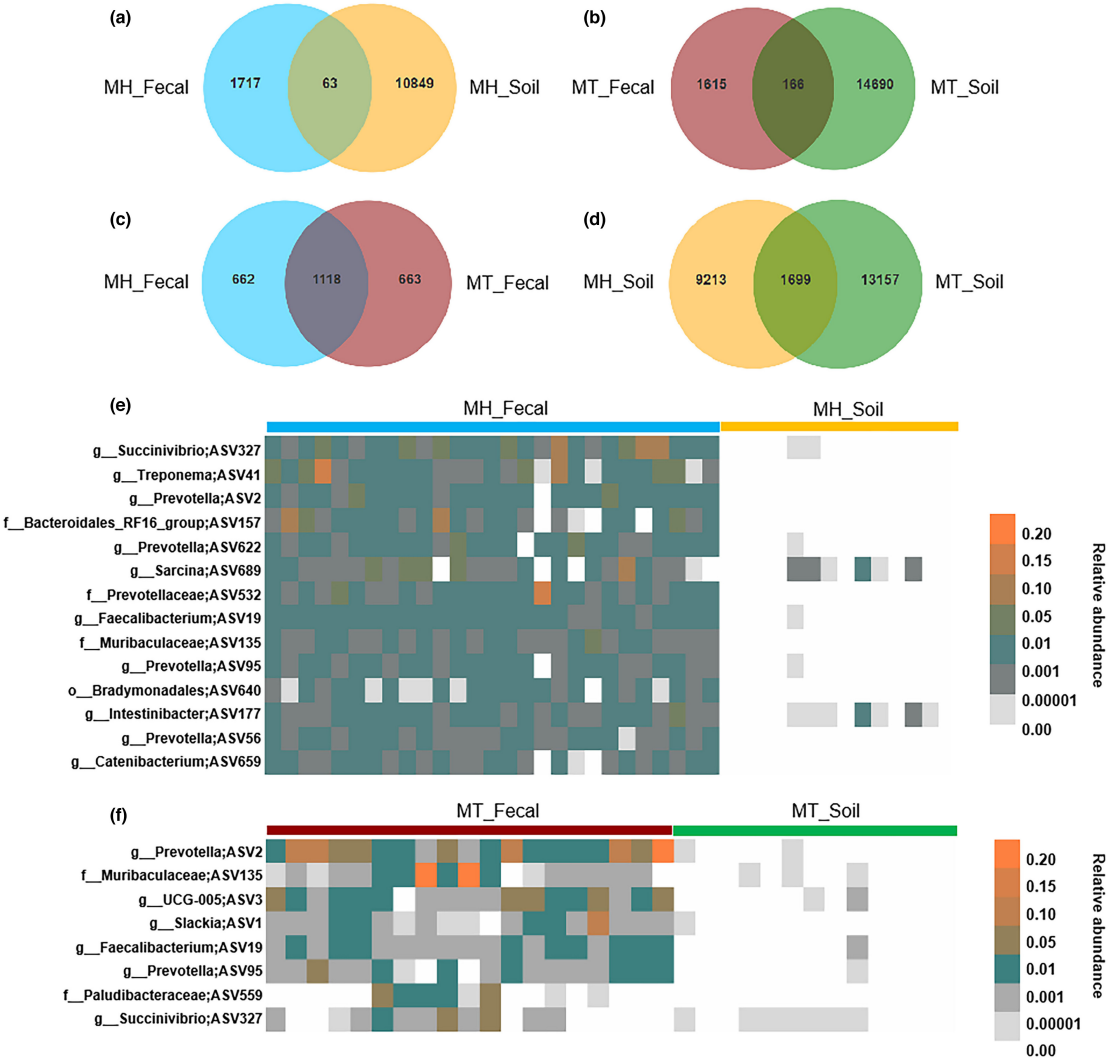
Shared ASVs between gut and soil bacterial microbiome. (a) Shared ASVs between gut and soil of Mt. Huangshan. (b) Shared ASVs between gut and soil of Mt. Tianhu. (c) Shared ASVs of the fecal bacterial microbiome between Mt. Huangshan and Mt. Tianhu. (d) Shared ASVs of the soil bacterial microbiome between Mt. Huangshan and Mt. Tianhu. (e) The distributions of abundant shared ASVs in fecal and soil samples of Mt. Huangshan. (f) The distributions of core abundant genera in fecal and soil samples of Mt. Tianhu.

Among the shared ASVs at each site, 14 were detected in Mt. Huangshan and 8 were detected at Mt. Tianhu with relative abundance greater than 1% of the corresponding gut bacterial microbiome. The taxonomic profiles, mean relative abundances, and occurrence rates of these ASVs are presented in Supplementary Table S3 and Table S4. We found that all the core abundant ASVs present in the gut bacterial microbiome (average relative abundance greater than 1% present on at least 80% of fecal samples) at each study site were present in the corresponding soil samples (Figure 4a,b). However, all the core ASVs abundant in the Tibetan macaque gut bacterial microbiome were rare bacterial taxa (low abundances: <1%, low occurrence rate: < 80%) in the corresponding soil samples. Notably, we found that none of the abundant ASVs (relative abundance >1%) of soil samples was shared by fecal samples at Mt. Huangshan or Mt. Tianhu. This suggests that the predominant bacterial groups from the soil were not likely to colonize the Tibetan macaques' gut (Figure 5).

## 4 DISCUSSION

We found that the two most known abundant phyla in Tibetan macaque guts are Firmicutes and Bacteroidota (with total mean relative abundances accounting for more than 84%). This result is consistent with the dominant phyla observed in the guts of other mammals, such as mice (Maurice et al., 2015), rabbits (Bäuerl et al., 2014), humans (Lozupone et al., 2012), and nonhuman primates (Springer et al., 2017). However, the dominant phyla in environmental soil are Proteobacteria, Acidobacteriota, Chloroflexi, and Actinobacteriota, indicating an obvious difference in gut bacterial community composition between the gut of Tibetan macaques and its living environmental soil. In addition, significant variation in alpha and beta diversity in the bacterial microbiome between Tibetan macaques' gut and corresponding habitat soil in each study site also was detected. In particular, the alpha diversity of fecal samples was significantly lower than that of corresponding soil samples in both sites, which supports the view that soil contains the most diverse and abundant group of microbes on Earth (Fierer, 2017; Trosvik et al., 2018).

Although the distance between Mt. Huangshan and Mt. Tianhu is only 10 kilometers, the divergence in beta diversities and community compositions of Tibetan macaques' gut bacterial microbiome between the two study groups were detected. The same was true of soil bacterial microbiome in both places. Tibetan macaques live in a matrilineal structured group of strictly linear hierarchy, and genetic similarities between individuals within the same group are greater than those between different groups (Li & Kappeler, 2020). In addition, the elevation of Mt. Tianhu group's activity area was 200 meters lower than that of Mt. Huangshan. Both sites share similar flora and fauna, and the main diet of the two groups includes leaves and grass, and to a lesser extent, fruits, flowers, roots, and insects. However, MH group is provisioned with corn, which is approximately one‐third of the daily food intake of the group. All these factors imply that the two study groups had distinct host genetics, diet resources, and habitat environments, which may contribute to the divergence of the gut bacterial microbiome (Linnenbrink et al., 2013; Muegge et al., 2011; Ochman et al., 2010), as well as the soil bacterial microbiome (Adamczyk et al., 2019). Notably, the alpha diversity MH group still maintains the same level as wild living individuals. A recent study has found that natural diets or releasing captive animals back into their natural habitat can help restore the alpha diversity of captive animals (Martinezmota et al., 2019; Zhou et al., 2018). This result supported that living in the wild and consuming a diverse diet is beneficial to maintaining the alpha diversity of the NHPs' gut microbiome (Barelli et al., 2020; Sun et al., 2021).

Furthermore, we also found that the core genera abundance in the Tibetan macaques' gut bacterial microbiome was rarely present in soil samples, which was similar to a previous study of the pikas' bacterial microbiome (Li et al., 2016). In fact, there is an opportunity for environmental bacteria of soil to flow into the gut of Tibetan macaques by contacting the topsoil, whereas the core abundant genera abundant in soil are almost undetectable in the gut of Tibetan macaques. Evidence in humans, NHPs, and lab mice have shown that host factors including sex, age, and immune responses are closely related to the community composition of the gut microbiome (Degnan et al., 2012; Kim et al., 2017). Additionally, other host factors such as physical, chemical, and bacterial barriers of the guts may limit competition and invasion of foreign microbes and therefore affect the colonization of environmental soil bacteria (Blum et al., 2019). Thus, the abundant bacteria enriched in soil samples are almost undetectable in the gut of Tibetan macaques and are likely in response to selective pressure from the host factors.

Finally, although there are some shared ASVs between gut and soil bacterial microbiome, the abundant ASVs in the guts of Tibetan macaques were rare bacterial taxa in the corresponding soil samples. This pattern suggests that the gut may select for rare but diverse environmental bacteria which have been proposed in the study of Pikas gut bacterial microbiome (Li et al., 2016). The same patterns also have been observed in Amphibians (*Batrachochytrium dendrobatidi*s) (Walke et al., 2014). It has been proposed that terrestrial and semi‐terrestrial mammals may have chances of exposure to fecal‐orally transmitted or soil‐borne gut microbes (Perofsky et al., 2019; Tung et al., 2015). Tibetan macaques often live in close physical proximity to one another and engage in unintentional sharing of fecal contaminants on the topsoil, which may colonize the gut while the host ingests food particles. However, whether these rare bacteria are permanent residents of the soil or a source of fecal contamination remains to be determined. Notably, we found that none of the abundant ASV of soil samples were shared by fecal samples at Mt. Huangshan or Mt. Tianhu. This suggests that the predominant bacterial groups from the soil were not likely to colonize the Tibetan macaques' gut.

## 5 CONCLUSIONS

In the present study, by evaluating the bacterial microbiome of wild Tibetan macaques and the related soil bacteria from their habitats, we could demonstrate that the Tibetan macaques' gut bacterial community showed manifest differences compared to the habitat soil bacteria. In addition, we found that most of the core genera abundance and ASVs in the Tibetan macaques' gut bacterial microbiome could be detected in the corresponding soil samples, but with low abundance. However, the core abundant genera abundant in soil are almost undetectable in the gut of Tibetan macaques. Although there are some shared ASVs between gut and soil bacterial microbiome, the abundant ASVs in the guts of Tibetan macaques were rare bacterial taxa in the corresponding soil samples. Notably, all the ASVs shared between guts and soil were present in the soil at relatively low abundance, whereas they were affiliated with diverse bacterial taxa. By linking the bacterial microbiome between Tibetan macaques' gut and its habitat soil, our findings suggest that the predominant bacterial groups of the soil were not likely to colonize the Tibetan macaques' gut, whereas the low‐abundance but diverse soil bacteria could be selected by the gut. Whether these rare and low‐abundant bacteria are permanent residents of the soil or a source of fecal contamination remains to be determined in future study.

### AUTHOR CONTRIBUTION

Xiaojuan Xu: Conceptualization (equal); Data curation (equal); Formal analysis (lead); Funding acquisition (supporting); Visualization (lead); Writing—original draft (lead); and Writing—review & editing (lead). Binghua Sun: Conceptualization (lead); Data curation (equal); Formal analysis (equal); Funding acquisition (supporting); Methodology (equal); Project administration (lead); and Writing—review & editing (supporting). Yingna Xia: Data curation (equal); Formal analysis (supporting); Methodology (supporting); Writing—original draft (supporting); and Writing—review & editing (supporting). All authors have read and agreed to the published version of the manuscript.

### FUNDING INFORMATION

The project was funded by the National Nature Science Funds of China (NSFC) (No. 32171488), Natural Science Foundation of Universities of Anhui Province (No. KJ2021A0923), Scientific Research Foundation for Advanced Talents of Hefei Normal University (No. 2020rcjj51), and International Collaborative Research Center for Huangshan Biodiversity and Tibetan Macaque Behavioral Ecology, Anhui University, Hefei, China (No. KF 200015).

### CONFLICT OF INTEREST

We have no conflict of interest to declare.

## Supporting information


Table S1
Table S2
Table S3
Table S4

## Data Availability

Raw data were submitted to the Sequence Read Archive of NCBI under the accession number PRJNA739400.
