# Supplementary material for: Linking the bacterial microbiome between gut and habitat soil of Tibetan macaque (Macaca thibetana)
Source: Ecol Evol. 2022 Sep 13;12(9):e9227. doi: 10.1002/ece3.9227 (PMC9471045; doi:10.1002/ece3.9227)
Supplement: Supplementary file 1 — Table S1 Table S2 Table S3 Table S4 [file ECE3-12-e9227-s001.docx]

Linking the bacterial microbiome between gut and habitat soil of Tibetan macaque (*Macaca thibetana*) - **Supplementary Material**

**Xiaojuan Xu^1^, Yingna Xia^2, 3^, Binghua Sun^2, 3^***

***Table S1*** Core abundant known genera in fecal and soil samples at Mt. Huangshan. Core taxonomy units were identified as those present on at least 80% of each sample types (fecal and soil) and at an average relative abundance of >1%. ^a^: Sample number, ^b^: mean relative abundance, ^b^: Occurrence rate.

| **Genus** | **MH_Fecal** | | |  | **MH_Soil** | | |
| --- | --- | --- | --- | --- | --- | --- | --- |
|  | S.N^a^ | M.R.A^b^ | O.R^c^ |  | S.N^a^ | M.R.A^b^ | O.R^c^ |
| *Prevotella* | 27 | **0.1552** | 100.00% |  | 14 | 0.0004 | 14.29% |
| *UCG-005* | 27 | **0.0552** | 100.00% |  | 14 | 0.0001 | 14.29% |
| *Treponema* | 27 | **0.0443** | 100.00% |  | 14 | 0.0001 | 14.29% |
| *Succinivibrio* | 27 | **0.0428** | 100.00% |  | 14 | 0.0001 | 14.29% |
| *Faecalibacterium* | 27 | **0.0414** | 100.00% |  | 14 | <0.0001 | 14.29% |
| *UCG-002* | 27 | **0.0269** | 100.00% |  | 14 | 0.0001 | 14.29% |
| *Sarcina* | 27 | **0.0256** | 85.20% |  | 14 | 0.0016 | 42.86 |
| *Blautia* | 27 | **0.0250** | 100.00% |  | 14 | 0.0000 | 14.29% |
| *Clostridium_sensu_stricto_1* | 27 | **0.0202** | 100.00% |  | 14 | 0.0053 | 64.27% |
| *Anaerostipes* | 27 | **0.0192** | 100.00% |  | 14 | < 0.0001 | 7.14% |
| *Alloprevotella* | 27 | **0.0152** | 100.00% |  | 14 | < 0.0001 | 14.29% |
| *Bifidobacterium* | 27 | **0.0151** | 85.20% |  | 14 | < 0.0001 | 7.14% |
| *Intestinibacter* | 27 | **0.0138** | 100.00% |  | 14 | 0.0012 | 50.00% |
| *Catenibacterium* | 27 | **0.0120** | 92.60% |  | 14 | 0.0000 | 0.00% |
| *Ruminococcus* | 27 | **0.0101** | 100.00% |  | 14 | < 0.0001 | 14.29% |
| *Acidibacter* | 27 | 0.0000 | 0.00% |  | 14 | **0.0154** | 100% |
| *Acidothermus* | 27 | 0.0000 | 0.00% |  | 14 | **0.0150** | 100% |
| *Bryobacter* | 27 | 0.0000 | 0.00% |  | 14 | **0.0138** | 100% |
| *Anaeromyxobacter* | 27 | 0.0000 | 0.00% |  | 14 | **0.0134** | 100% |
| *Candidatus_Udaeobacter* | 27 | 0.0000 | 0.00% |  | 14 | **0.0132** | 93% |
| *Bradyrhizobium* | 27 | 0.0000 | 0.00% |  | 14 | **0.0100** | 100% |

***Table S2*** Core abundant known genera in fecal and soil samples at Mt. Tianhu. Core taxonomy units were identified as those present on at least 80% of each sample types (fecal and soil) and at an average relative abundance of >1%. ^a^: Sample number, ^b^: mean relative abundance, ^b^: Occurrence rate.

| **Genus** | **MT_Fecal** | | |  | **MT_Soil** | | |
| --- | --- | --- | --- | --- | --- | --- | --- |
|  | S.N^a^ | M.R.A^b^ | O.R^c^ |  | S.N^a^ | M.R.A^b^ | O.R^c^ |
| *Prevotella* | 19 | **0.1516** | 100.00% |  | 13 | 0.0004 | 69.23% |
| *UCG-005* | 19 | **0.0727** | 100.00% |  | 13 | 0.0002 | 61.54% |
| *Faecalibacterium* | 19 | **0.0378** | 100.00% |  | 13 | 0.0001 | 38.46% |
| *Treponema* | 19 | **0.0289** | 100.00% |  | 13 | 0.0001 | 30.77% |
| *UCG-002* | 19 | **0.0245** | 100.00% |  | 13 | 0.0001 | 53.85% |
| *Subdoligranulum* | 19 | **0.0236** | 100.00% |  | 13 | < 0.0001 | 15.38% |
| *Blautia* | 19 | **0.0228** | 94.74% |  | 13 | 0.0001 | 46.15% |
| *Succinivibrio* | 19 | **0.0152** | 100.00% |  | 13 | 0.0001 | 53.85% |
| *Slackia* | 19 | **0.0147** | 94.74% |  | 13 | 0.0000 | 0.00% |
| *Roseburia* | 19 | **0.0126** | 100.00% |  | 13 | < 0.0001 | 23.08% |
| *Ruminococcus* | 19 | **0.0105** | 100.00% |  | 13 | 0.0000 | 0.00% |
| *Bryobacter* | 19 | 0.0000 | 0.00% |  | 13 | **0.0483** | 100.00% |
| *Acidothermus* | 19 | < 0.0001 | 10.53% |  | 13 | **0.0384** | 100.00% |
| *Conexibacter* | 19 | 0.0000 | 0.00% |  | 13 | **0.0360** | 100.00% |
| *Candidatus_Solibacter* | 19 | 0.0000 | 0.00% |  | 13 | **0.0353** | 100.00% |
| *Mycobacterium* | 19 | 0.0000 | 0.00% |  | 13 | **0.0236** | 100.00% |
| *Acidibacter* | 19 | 0.0000 | 0.00% |  | 13 | **0.0152** | 100.00% |

***Table S3*** Abundant ASVs of fecal and soil samples in Mt. Huangshan. Core taxonomy units were identified as those present on at least 80% of each sample types (fecal and soil) and at an average relative abundance of >1%. ^a^: Sample number, ^b^: mean relative abundance, ^b^: Occurrence rate.

| **ASV ID** | **Taxonomy Units** | **MH_Fecal** | | |  | **MH_Soil** | | |
| --- | --- | --- | --- | --- | --- | --- | --- | --- |
|  |  | S.N^a^ | M.R.A^b^ | O.R^c^ |  | S.N^a^ | M.R.A^b^ | O.R^c^ |
| ASV327 | *g__Succinivibrio* | 27 | **0.0425** | 100.00% |  | 14 | 0.0001 | 14.29% |
| ASV41 | *g__Treponema* | 27 | **0.0329** | 100.00% |  | 14 | 0.0000 | 0.00% |
| ASV2 | *g__Prevotella* | 27 | **0.0265** | 96.55% |  | 14 | 0.0000 | 0.00% |
| ASV157 | f__Bacteroidales_RF16_group | 27 | **0.0260** | 89.66% |  | 14 | 0.0000 | 0.00% |
| ASV622 | *g__Prevotella* | 27 | **0.0236** | 96.55% |  | 14 | < 0.0001 | 14.29% |
| ASV689 | *g__Sarcina* | 27 | **0.0218** | 86.21% |  | 14 | 0.0015 | 42.86% |
| ASV532 | f__Prevotellaceae | 27 | **0.0206** | 100.00% |  | 14 | 0.0000 | 0.00% |
| ASV19 | *g__Faecalibacterium* | 27 | **0.0180** | 100.00% |  | 14 | < 0.0001 | 7.14% |
| ASV135 | f__Muribaculaceae | 27 | **0.0157** | 100.00% |  | 14 | 0.0000 | 0.00% |
| ASV95 | *g__Prevotella* | 27 | **0.0154** | 96.55%% |  | 14 | < 0.0001 | 7.14% |
| ASV640 | o__Bradymonadales | 27 | **0.0146** | 96.55%% |  | 14 | 0.0000 | 0.00% |
| ASV177 | *g__Intestinibacter* | 27 | **0.0127** | 100.00% |  | 14 | 0.0011 | 50.00% |
| ASV56 | *g__Prevotella* | 27 | **0.0124** | 100.00% |  | 14 | 0.0000 | 0.00% |
| ASV659 | *g__Catenibacterium* | 27 | **0.0119** | 93.10% |  | 14 | 0.0000 | 0.00% |

***Table S4*** Abundant ASVs of fecal and soil samples in Mt. Tianhu. Core taxonomy units were identified as those present on at least 80% of each sample types (fecal and soil) and at an average relative abundance of >1%. ^a^: Sample number, ^b^: mean relative abundance, ^b^: Occurrence rate.

| **ASV ID** | **Taxonomy Units** | **MT_Fecal** | | |  | **MT_Soil** | | |
| --- | --- | --- | --- | --- | --- | --- | --- | --- |
|  |  | S.N^a^ | M.R.A^b^ | O.R^c^ |  | S.N^a^ | M.R.A^b^ | O.R^c^ |
| ASV2 | *g__Prevotella* | 19 | **0.0604** | 100.00% |  | 13 | < 0.0001 | 15.38% |
| ASV135 | f__Muribaculaceae | 19 | **0.0416** | 89.47% |  | 13 | < 0.0001 | 23.08% |
| ASV3 | *g__UCG-005* | 19 | **0.0280** | 94.74% |  | 13 | 0.0001 | 15.38% |
| ASV1 | *g__Slackia* | 19 | **0.0147** | 94.74% |  | 13 | < 0.0001 | 7.69% |
| ASV19 | *g__Faecalibacterium* | 19 | **0.0127** | 100.00% |  | 13 | < 0.0001 | 7.69% |
| ASV95 | *g__Prevotella* | 19 | **0.0125** | 89.47% |  | 13 | < 0.0001 | 7.69% |
| ASV559 | f__Paludibacteraceae | 19 | 0.0114 | 42.11% |  | 13 | 0.0000 | 0.00% |
| ASV327 | *g__Succinivibrio* | 19 | 0.0112 | 57.89% |  | 13 | 0.0001 | 53.85% |
